# Salvage Radiation for Pelvic Relapse after Surgically Treated Endometrial Cancer

**DOI:** 10.3390/cancers13061367

**Published:** 2021-03-18

**Authors:** Kristina Lindemann, Elisabeth Smogeli, Milada Cvancarova Småstuen, Kjersti Bruheim, Jone Trovik, Terje Nordberg, Gunnar B. Kristensen, Henrica M. J. Werner, Esten Nakken

**Affiliations:** 1 Department of Gynecological Oncology, Division of Cancer Medicine, Oslo University Hospital, PB 4953 Nydalen, 0424 Oslo, Norway; 2 Institute of Clinical Medicine, Faculty of Medicine, University of Oslo, P.O. Box 1171 Blindern, 0318 Oslo, Norway; esmogeli@mac.com; 3 Department of Nursing and Health Promotion, Faculty of Health Sciences, Oslo Metropolitan University, 0166 Oslo, Norway; milasm@oslomet.no; 4 Department of Oncology, Division of Cancer Medicine, Oslo University Hospital, PB 4953 Nydalen, 0424 Oslo, Norway; UXKJUH@ous-hf.no (K.B.); uxnakk@ous-hf.no (E.N.); 5 Centre for Cancer Biomarkers, Department of Clinical Science, University of Bergen, 5020 Bergen, Norway; jone.trovik@uib.no; 6 Department of Obstetrics and Gynecology, Haukeland University Hospital, 5053 Bergen, Norway; 7 Department of Oncology, Haukeland University Hospital, 5021 Bergen, Norway; terje.nordberg@helse-bergen.no; 8 Institute for Cancer Genetics and Informatics, Department of Oncology, Division of Cancer Medicine, Oslo University Hospital, 0424 Oslo, Norway; gunnar.b.kristensen@gmail.com; 9 Department of Obstetrics and Gynecology, Grow School for Oncology and Developmental Biology, Maastricht University Medical Centre, P.O. Box 5800, 6202 AZ Maastricht, The Netherlands; erica.werner@mumc.nl

**Keywords:** endometrial cancer, pelvic relapse, vaginal relapse, outcome, salvage radiation

## Abstract

**Simple Summary:**

This multicenter retrospective study aimed to describe the outcomes of patients with endometrial cancer after central pelvic/vaginal relapse treated with radical radiotherapy (RT). We included 139 patients with a median follow-up time of 6.66 years. Patients were treated with external beam radiotherapy to elective pelvic lymph-node regions and boost to the pelvic tumor. During follow-up, 55 (39.6%) patients developed a second relapse, the majority (75%) with disease sites outside the radiation field. Risk group at primary diagnosis and type of boost administration were independent predictors of progression-free and overall survival. Five-year overall survival for the whole cohort was 68% (95% CI: 59–75%). The majority of isolated pelvic recurrences in RT-naive women with EC can be successfully salvaged by RT but survival in high-risk patients remains suboptimal. Individualizing of adjuvant treatment in first line and better treatment alternatives at relapse are important to ultimately improve survival.

**Abstract:**

(1) *Background*: This study evaluated the clinical outcome after salvage radiotherapy for first pelvic relapse after endometrial cancer (EC). (2) *Methods*: This multicenter retrospective study included EC patients with first central pelvic relapse without lymph node involvement treated with curative intent. Progression-free (PFS) and overall survival (OS) were calculated with the Kaplan–Meier method and possible predictive factors for risk of relapse and mortality were identified using the Cox model. (3) *Results*: We included 139 patients with median EQD2 (Equivalent Dose in 2 Gy fractions) to the clinical target volume of 70.0 Gy. During follow up of median 6.66 years, 39.6% patients developed a second relapse. Risk group classification at primary diagnosis based on histology, grading and FIGO stage and how the pelvic tumor boost was administered were independently associated with PFS and OS. Five-year OS was 68% (95% CI (59–75)) for the whole cohort. Five-year OS was 88% (95% CI (75–94)), 72% (95% CI (55–84)) and 38% (95% CI (15–60)) for the stage I low-, intermediate- and high-risk group, respectively. (4) *Conclusions*: The majority of central pelvic recurrences in RT-naive EC women can be successfully salvaged with radiotherapy. However, survival in patients with high-risk disease remains poor and warrants a more individualized approach to optimize outcome.

## 1. Introduction

Endometrial cancer (EC) is the most common malignancy of the female genital tract in affluent societies [1]. It has a predominantly favorable outcome mainly due to the fact that the majority of cases are diagnosed at an early stage because of abnormal vaginal bleeding. Optimal adjuvant treatment after surgery is still a matter of debate and is currently tailored to the presence of risk factors including the stage of disease, depth of myometrial invasion, grade of the tumor, lymphovascular invasion (LVSI) and lymph-node metastasis at the time of primary diagnosis [2]. Women with stage I (early) endometrial cancer have a low risk of recurrence of their disease as less than 10% of women treated with surgery alone will recur after surgery [3,4]. This risk is significantly higher for women with high-risk factors including poorly differentiated or serous tumors and deep invasion of the myometrium. External beam radiotherapy (EBRT) after surgery reduces the risk of pelvic recurrence but does not reduce the risk of death [5,6,7,8]. EBRT may also cause considerable treatment-related short- and long-term side effects [9,10]. Vaginal brachytherapy (VBT) has been shown to be a less toxic treatment choice when aiming for a reduction in vaginal relapses. In the Post Operative Radiation Therapy in Endometrial Carcinoma (PORTEC)-2 trial in intermediate-risk patients, there was no statistically significant difference in the locoregional recurrence rate (5% vs. 3%) between VBT and EBRT or survival, but significantly less bowel- and genito-urinary side effects [11,12]. In the following PORTEC III trial in high-risk patients, the addition of chemotherapy to radiation yielded longer survival rates of modest effect size in a post hoc survival analysis [13,14], but there is still no convincing benefit of the combination of these two modalities when compared to chemotherapy alone [15].

The lack of survival benefit after radiotherapy (RT) and the mounting data that locoregional relapses can potentially be treated with salvage radiotherapy with EBRT and VBT has led to the omission of adjuvant radiotherapy in the front line of most patients with endometrial cancer in Nordic countries. This strategy is further supported by the PORTEC-1 trial reporting a complete response rate of 87% in RT-naïve women treated with salvage RT for an isolated vaginal recurrence [16]. A population-based study from Denmark further confirmed that the omission of RT in intermediate risk endometrial cancer patients had no detrimental effect on survival [17]. Since the conducting of these studies, a more refined classification of EC incorporating molecular characteristics has been established [18,19], which adds prognostic information also independently of known histopathologic risk groups [20].

The aim of this multicenter retrospective study was to describe the outcomes of patients with endometrial cancer after central pelvic/vaginal relapse treated with radical RT at two university hospitals in Norway. We also explored possible predictive risk factors for second relapse and mortality.

## 2. Results

We included 139 patients, 102 at Oslo University Hospital and 37 from Haukeland University Hospital with a median age of 71.3 (range 43.9 to 88.8) years at the time of pelvic relapse. Median follow-up after pelvic recurrence was 6.66 (0.12 to 12) years.

The majority of patients had stage I disease at primary diagnosis and the distribution according to risk groups is displayed in Table 1. All patients were radiotherapy naïve; 28 (20%) had received adjuvant chemotherapy at primary diagnosis. There was no statistically significant difference in the distribution of baseline characteristics across the two participating sites.

All patients were treated with pelvic external beam radiotherapy. Median EQD2 (Equivalent Dose in 2 Gy fractions using an α/β of 10 for tumor) to the clinical target volume defined for the local tumor relapse was 70.0 Gy ranging from 38.9 to 83.8 Gy. In total, 115 patients (83%) received an EQD2 of ≥67 Gy; 136 (94%) received EBRT against elective pelvic lymph-node regions with median dose of 50.0 Gy (range 28–50 Gy). For the pelvic tumor boost, 53 patients (38%) received EBRT with a simultaneous integrated or sequential boost; in 47 patients (34%) the boost was combined (EBRT and VBT), while in 39 patients (28%), the boost was given as VBT only.

During follow-up, 55 (39.6%), patients developed a second relapse. For 46 of those, localization of the second relapse was documented. Twelve out of those 46 patients developed a second relapse inside the irradiated field alone. For the remaining patients, the second relapse was either outside (25/46) or both inside and outside the radiotherapy field (9/46). We studied the prevalence of the second relapse separately in stage I risk groups. In patients with low-risk disease at primary diagnosis, 20% (10/49) had a second relapse following salvage treatment. In the intermediate-risk group, 39% (14/36), and in the high-risk group, 69% of the patients (11/16) developed a second relapse. Thus, the risk of relapse after salvage radiotherapy was significantly different depending on risk group (*p* = 0.001).

**Figure 1:**
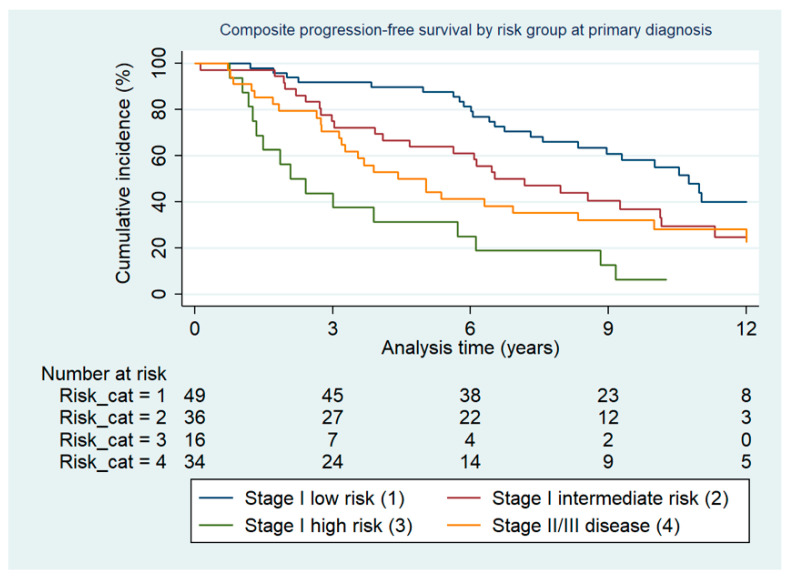
Composite progression-free survival by risk group at primary diagnosis.

The cumulative incidence of the composite endpoint progression-free survival in risk groups at primary diagnosis is depicted in Figure 1.

**Figure 2:**
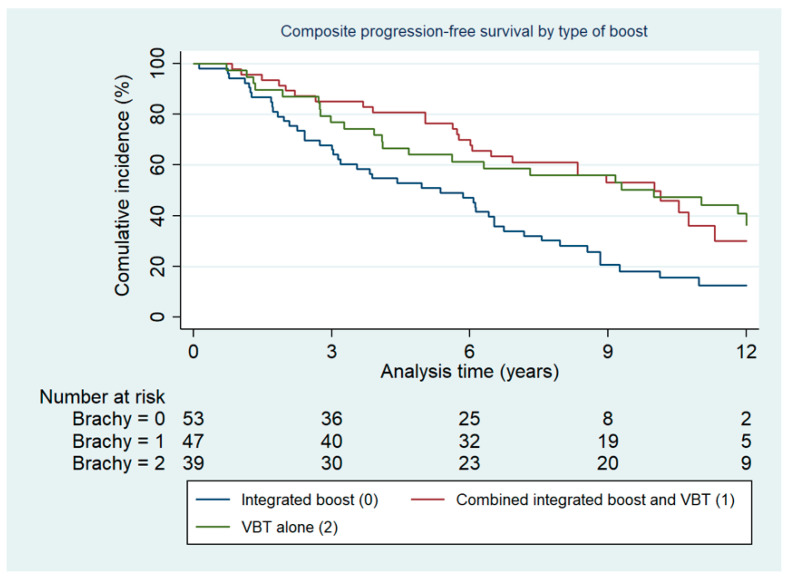
Composite progression-free survival by type of boost administration.

There was a statistically significantly shorter cumulative progression-free survival dependent on risk group at primary diagnosis (*p* < 0.001). There was no statistically significant difference in terms of localization of relapse (inside vs. outside vs. both) between risk groups (*p* = 0.496). There were more second relapses after EBRT with an integrated EBRT boost administered: 29/53 (55%) compared to 13/47 (28%) and 13/39 (33%) when the boost was either given combined with VBT or as VBT alone, respectively (*p* = 0.014). The cumulative incidence of the composite endpoint progression-free survival in groups by type of boost administration is depicted in Figure 2.

### 2.1. Survival

**Figure 3:**
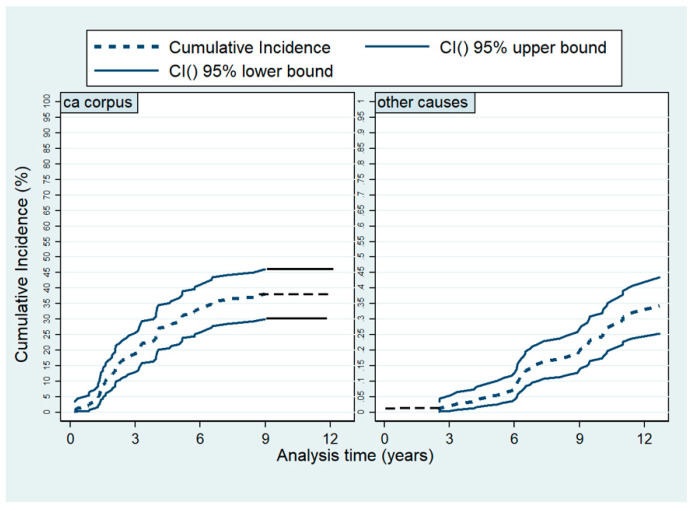
Cumulative overall mortality by causes of death.

After salvage radiotherapy, the cumulative incidence of being relapse free and alive at 5 years was 65% (95% CI (56–72)%) and after 10 years, 37% (95% CI (27–45)%). During the entire follow-up of median 6.66 (0.12 to 12) years, 63% (*n* = 88) of the study population died. Of those, 51 (58%) died due to endometrial cancer; 37 (42%) died of other causes. Five-year overall survival was 68% (95 %CI (59–75)%) and 10-year OS was 38% (95% CI (29–46)%). The 5-year OS estimates were 88% (95% CI (75–94)%), 72% (95% CI (55–84)%) and 38% (95% CI (15–60)%) in the stage I low-, intermediate- and high-risk group, respectively. We have decomposed the overall survival dividing causes of death into two groups, death due to endometrial or due to other causes, and used competing risk methodology to depict the cumulative incidences as illustrated in Figure 3.

### 2.2. Predictive Factors for Survival: Relative Risk of Second Relapse

In univariate analyses, type of boost administration and risk group at primary diagnosis were significantly associated with higher risk of second relapse, while age and EQD2 of equal or higher than 67 Gy were not. In multivariate analysis, both the type of boost administration and risk group remained independent predictive factors for second relapse and death. Compared to patients with low-risk disease at primary diagnosis, patients with stage I high-risk disease had significantly increased risk of second relapse (HR 5.60, 95% CI (2.36–13.27)). However, this risk was not significantly increased for patients with intermediate risk (HR 1.93, 95% CI (0.86–4.35)). In patients with stage II/III disease, the risk of second relapse was even higher (HR 3.31, 95% CI (1.53–7.18)). Patients who received at least part of the boost as VBT had a significantly reduced risk for second relapse (HR 0.41; 95% CI (0.21–0.79)) for the combined boost; HR 0.48, 95% CI (0.25–0.93) for VBT).

### 2.3. Predictive Factors for Survival: Overall Mortality Risk

In univariate analyses, age, risk-group at primary diagnosis and type of boost administration were significantly associated with overall survival (Table 2). However, dose to the clinical target volume and localization of second relapse were not significantly associated. In multivariate regression analysis, patients with stage I high-risk disease at primary diagnosis had a significantly higher mortality risk compared to patients with low-risk of disease (HR 3.68, 95% CI (1.83–7.42)) (Table 2). For patients with intermediate-risk, this risk increase was not statistically significant (HR 1.27, 95% CI (0.69–2.36)). Compared with patients in whom the boost was given as EBRT alone, patients who received part of the boost as VBT had a significantly reduced risk of death (HR 0.50, 95% CI (0.29–0.86) in patients with combined VBT + EBRT boost; HR 0.45, 95% CI (0.25–0.80) and in patients with VBT only).

## 3. Discussion

The majority of isolated pelvic recurrences in radiotherapy-naïve women with endometrial cancer can be successfully treated with salvage radiotherapy. Five-year OS was 68% (95% CI (60–75)) for the whole cohort, which is in line with other reports in series of patients who had not undergone radiotherapy as adjuvant treatment [3]. Survival rates in patients who had undergone radiotherapy in first line have been reported to be as low as 43% [16]. Risk classification at primary diagnosis and how the boost to the pelvic tumor was administered (EBRT alone, VBT/EBRT combined or with VBT alone) were independent risk factors for second relapse and survival. Despite the high cure rates in patients with low-risk disease at primary diagnosis, the more modest survival rates in the intermediate-risk group as well as the rather poor 5-year survival in patients with stage I high-risk and stage II/III disease warrant further improvement and individualization of treatment in these patients.

This is the largest multicenter study of patients with isolated pelvic relapses after surgically treated endometrial cancer. All patients were radiotherapy-naïve in line with the Norwegian national treatment guidelines [21] where adjuvant radiotherapy is omitted in front line treatment of patients with endometrial cancer. As expected, the majority of patients were of low/intermediate risk at primary diagnosis, highlighting the rather high risk of distant relapse in patients with high-risk disease, as shown in a published series of early stage high risk patients from our institution [22]. We demonstrated the prognostic significance of risk classification at primary diagnosis based on grading, histology and FIGO stage. The reported 5-year OS of 68% needs to be interpretated in light of the fact that 37% of the included patients had high-risk stage I or stage II–III disease at primary diagnosis. In the published series of patients with pelvic recurrences after surgically treated endometrial cancer, 5-year overall survival rates of 75–84% [23,24,25] have been reported. These high survival rates apply to patients with predominantly low- and intermediate risk features, and they drop down to 25–43% in studies including a higher proportion of high risk or advanced disease [26,27]. Salvage rates are also higher in radiotherapy-naïve patients than in patients who have received postoperative radiotherapy [16]. Together with the reported low rate of loco-regional relapses, especially in early stage endometrioid endometrial cancer [3,4], this supports the strategy in Nordic countries of saving the administration of radiotherapy for the salvage treatment of isolated pelvic relapse instead of its administration in first line to prevent loco-regional relapse. Our reported pelvic control rate of 25% developing a second relapse inside the previous irradiated field is in line with other studies [25]. The superior outcome of patients where the boost was administered as VBT alone or as VBT combined with EBRT might at least partly be due to residual confounding. Larger tumors with poorer prognosis might be overrepresented in the group of patients who received an external boost only, while patients with low volume disease may have been treated with VBT.

Patients with high-risk stage I disease or more advanced disease still represent a therapeutic challenge. Their outcome after chemotherapy alone, especially in patients with lymph node-positive disease, needs further improvement [15,22] and also, the poorer survival rates after central pelvic relapse warrant further optimization of their treatment. Improved pelvic control rate after adjuvant pelvic radiation is well-documented but not associated with a survival benefit in patients with stage I and II disease, not even in the high-risk group [28,29]. In advanced disease, results are still conflicting. The PORTEC 3 study showed a benefit in progression-free and overall survival when radiotherapy was combined with systemic chemotherapy [13] and molecular features such as p53 have been demonstrated to be a prognostic biomarker also in patients with high-risk disease [30]. However, in the GOG 258 study, including patients with stage III and IV only, the combination of these two modalities did not increase survival when compared to chemotherapy alone [15]. We need to acknowledge that the poor survival in these patients is driven by the high risk of distant disease and that the routine administration of upfront pelvic radiation comes at a price of considerable short- and long-term toxicity for these often elderly and comorbid patients. Current studies explore the addition of immunotherapy both in first line (NCT04634877) and recurrent setting (NCT03981796) but understanding the predictive markers such as MMR status for treatment response and tolerability will be crucial for patient selection and individualized treatment.

Our study is the largest study of patients with isolated pelvic relapse to date. The two participating sites cover a catchment area of around 4 million inhabitants, and all relapses in these regions are referred to these two centers. Thus, the cohort represents a population-based sample of patients with central pelvic/vaginal relapse without lymph node involvement. The lack of information on molecular classifiers and other prognostic factors such as LVSI is a limitation of our study as well as the absence of detailed information on the pelvic extension of the disease at the time of relapse. By excluding patients with stage IV disease as well as patients with pelvic lymph node involvement, we are still confident that the population is representative for patients with central pelvic and vaginal relapse.

## 4. Materials and Methods

### 4.1. Patients and Follow-Up

This is a retrospective study of a cohort patients with endometrial cancer treated with curative intent for their first histologically verified central pelvic or vaginal relapse without lymph node involvement at the Department of gynecological oncology, Oslo University Hospital (The Norwegian Radium Hospital) or the Department of Obstetrics and Gynecology, Haukeland University Hospital, Norway. At Oslo University Hospital, patients treated between 2006 and 2011 were identified in a validated quality assurance database, providing detailed information on the primary diagnosis, surgical treatment, adjuvant treatment, time and localization of relapse. Details on the radiotherapy treatment administered were available through the hospital’s oncology management system Mosaiq (Elekta, Sunnyvale, CA, USA). Patients treated at Haukeland University Hospital were identified in the MoMaTEC (Molecular Markers in the Treatment of Endometrial Cancer study (NCT00598845) database which was a prospective population-based series of patients with endometrial cancer including patients from 2001 to 2015. Details on the radiotherapy administered were available through the hospital’s oncology management system Varian Medical Systems (Palo Alto, CA, USA) and the patients’ electronic records. The radiotherapy applied was either given with a four-field technique or with intensity-modulated radiation therapy (IMRT). For the patients receiving brachytherapy as a boost technique, this was given with high-dose rate (HDR). For more superficial recurrences, the treatment was delivered with a vaginal cylinder and the prescribed dose applied to 5 mm below the mucosal surface unless CT and/or gynecological examination indicated otherwise. The more advanced tumors received a combination of intracavitary and interstitial (IC/IC) brachytherapy. Patients were excluded if they had undergone pelvic radiotherapy as part of their primary treatment, stage IV disease at the time of diagnosis or metastatic pelvic lymph nodes at the time of relapse. A CT (computer tomography) scan was available for all patients to exclude extra pelvic disease. Stage of disease at initial surgery was recoded to match the International Federation of Gynecology and Obstetrics (FIGO) 2009 revised staging [31]. Individual survival data were available through linkage to Statistics Norway.

Patients were categorized according to their risk-group at primary diagnosis [21]. Patients with endometrioid endometrial cancer G1 or G2 and stage IA were classified as low-risk. Patients with endometrioid endometrial cancer G1 or G2 and stage IB or endometrioid endometrial cancer (EEC) G3 and stage IA were classified as intermediate-risk. Patients with endometrioid endometrial cancer G3 and stage IB or stage I type II tumors (non-endometrioid EC) were classified as high-risk. Patients with stage II and III disease were categorized as a fourth subgroup.

### 4.2. Statistical Analyses

Continuous variables were described with median and range. Categorical variables were presented with counts and proportions. Crude cumulative incidences were calculated with the reverse Kaplan–Meier method. For progression-free survival (PFS), follow-up time was calculated from the date of start of radiotherapy until the date of second relapse, date of death from any cause or end of follow-up, 31 May 2019, whichever occurred first. Due to the small number of patients with follow up beyond 12 years, PFS time was truncated at 12 years. When depicting the cumulative incidences of relapse in selected groups, we used a composite endpoint for progression-free survival to avoid immortal time bias. PFS was defined as occurrence of either second relapse or death. For overall survival (OS), follow-up time was calculated from the start of radiotherapy until date of death from any cause or end of follow-up, whichever occurred first. Crude survival curves were depicted with the Kaplan–Meier method. We calculated 5- and 10-year progression-free survival (using the composite endpoint defined above) and 5- and 10-year overall survival (OS) with 95% confidence intervals (CI). For OS survival analyses, follow up time was truncated at 12 years. Crude differences between groups were assessed using log-rank test. Possible predictive factors for risk for relapse and overall mortality were identified using multivariate Cox models. Unlike progression-free survival, the endpoint used to model risk for relapse was the second relapse only. Events such as death and end of follow up were treated as censored. Variables that reached *p*-values < 0.05 in univariate analyses were entered into the final multivariate model. Visual inspection of Schoenfeld’s residuals was performed to test if the assumption of proportional hazards was fulfilled. The results are expressed as hazard ratios (HR) with 95% confidence intervals (CI).

To decompose the composite endpoint of second relapse and death, we used competing risk methodology and depicted the cumulative incidences of second relapse and death separately using the Fine and Gray approach. The cumulative incidences of selected causes of death were also depicted using the Fine and Gray approach.

The analyses were performed using IBM SPSS version 23 (SPSS, Chicago, IL, USA) and the STATA statistical package, version 11.0, (Stata Corp LP, TX, USA).

## 5. Conclusions

In conclusion, our study adds to the evidence that the majority of central pelvic/vaginal relapses in radiation-naïve patients with endometrial cancer can be successfully treated with salvage radiotherapy. The retrospective data presented here reflect the treatment policy of the participating institutions at a time when molecular classifiers were not part of the routine diagnostic assessment. This change is practice is needed to individualize treatment such as recommended in the recently published joined statement by ESGO/ESTRO/ESP [32]. Future research efforts should focus on patients with early stage high-risk and advanced stage disease as these remain a therapeutic challenge also at the time of pelvic relapse. Future research on predictive markers as well as incorporating prognostic molecular classifiers are needed to personalize adjuvant treatment and improve survival at relapse.

## Figures and Tables

**Table 1:** Baseline characteristics of 139 patients with isolated pelvic relapse.

Variable	Value	Value
Age (mean/range)	71.3 years	43.9 to 88.8 years
Stage at initial diagnosis	*n*	%
IA	71	51.08%
IB	34	24.46%
II	16	11.51%
III	18	12.95%
Histology at primary diagnosis		
Endometrioid adenocarcinoma	111	79.86%
Serous/clear cell adenocarcinoma	20	14.39%
Other *	6	4.32%
Risk group at primary diagnosis for patients with stage I disease **		
Stage I low-risk	49	36.30%
Stage I intermediate-risk	36	26.67%
Stage I high-risk	16	11.85%
Previous treatment		
Hysterectomy/BSOE	139	100%
Pelvic lymphadenectomy	81	58.27%
Adjuvant chemotherapy	28	20.14%

* Carcinosarcoma (*n* = 3), adenosquamous (*n* = 2), unspecified (*n* = 1). ** Four patients with stage I disease could not be categorized due to missing information on grading.

**Table 2:** Multivariate Cox regression analyses for risk of second relapse and death.

Variable	Association with Risk of Relapse			Association with Risk of Death		
	HR	95% CI	*p*-Value	HR	95% CI	*p*-Value
Age	*	*	*	1.03	1.00–1.05	0.04
Stage I low risk (reference)	1.0			1.0		
Stage I intermediate risk	1.93	0.86–4.35	0.112	1.27	0.69–2.36	0.44
Stage I high risk	5.60	2.36–13.27	<0.001	3.68	1.83–7.42	<0.01
Stage II–III	3.31	1.52–7.18	0.002	2.34	1.30–4.21	0.01
External boost only (reference)	1.0			1.0		
Combined boost	0.41	0.21–0.79	0.008	0.50	0.29–0.86	0.01
VBT	0.48	0.25–0.93	0.030	0.45	0.25–0.80	0.01

* Not significant in univariate analysis and therefore not included in the multivariate model.

## Data Availability

The data presented in this study are available on request from the corresponding author. The data are not publicly available due to restrictions by the data protection office at the participating sites.
